# Seeing Things Differently

**DOI:** 10.3201/eid2002.AC2002

**Published:** 2014-02

**Authors:** Sharon Bloom, Alexandra M. Levitt

**Affiliations:** Centers for Disease Control and Prevention, Atlanta, Georgia, USA (S. Bloom); and Centers for Disease Control and Prevention, New York, New York, USA (A.M. Levitt)

**Keywords:** zoonoses, zoonotic pathogens, cover art, Seeing Things Differently, Nellie Mae Rowe, Picking Cotton, African American history month, self-taught art

**Figure:**
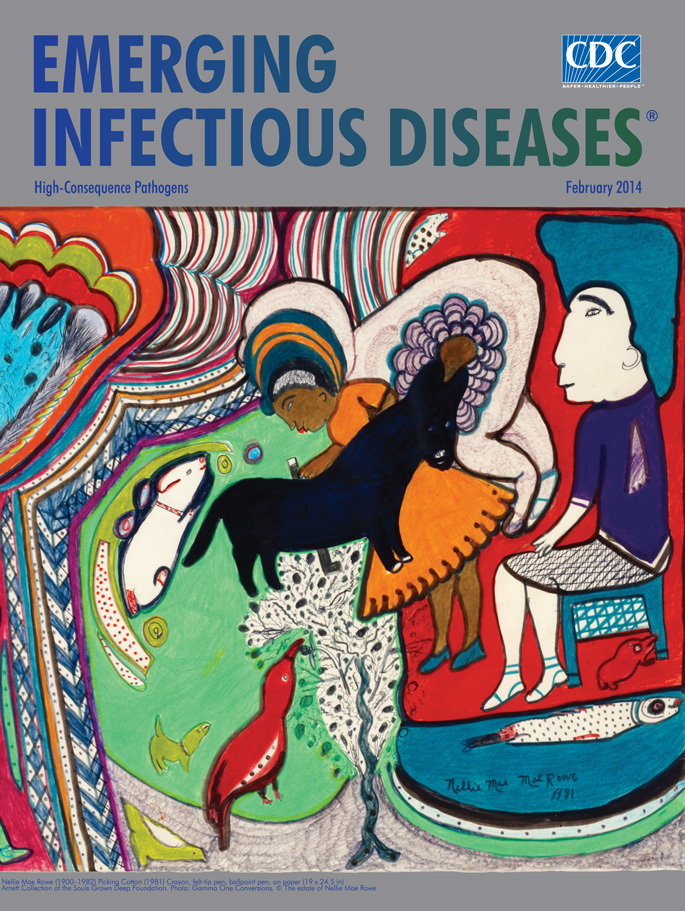
**Nellie Mae Rowe (1900–1982) *Picking Cotton* (1981) Crayon, felt-tip pen, ballpoint pen, on paper (19 × 24.5 in)** Arnett Collection of the Souls Grown Deep Foundation. Photo: Gamma One Conversions. © The estate of Nellie Mae Rowe

“You can draw a mule, dog, cat, or a human person, I’m going to draw it different. ‘Cause you always see things different. Each person’s different.” —Nellie Mae Rowe

*Picking Cotton,* by Nellie Mae Rowe (1900–1982), is a work of folk art that captures Rowe’s experiences as an African American woman living in rural Georgia, United States. Nellie Mae Rowe was born on July 4, 1900, as the ninth of ten children. The family lived on a rented farm 20 miles south of Atlanta. Her father, a farmer and blacksmith, was born a slave in 1854; her mother, born the year after President Lincoln issued the Emancipation Proclamation (1864), was a gospel singer and talented quilter.

Early childhood was pleasant for Nellie Mae, who had a natural affinity for drawing and making dolls. However, after 4 years of elementary school, she was put to work on the family farm and in the neighbors’ fields. At 16, she married Ben Wheat, and in 1930, the couple moved to Vinings, Georgia. Wheat died in 1936, and after a year, Nellie Mae married an older widower, Henry Rowe.

During and after marriage, Nellie Mae Rowe worked as a domestic servant. When her second husband died in 1948, she felt liberated to resurrect her early creative urges. She converted her 4-room Vinings home into her “playhouse”—decorating all available space inside and out in the yard with dolls, drawings, and other fanciful creations. A devout Christian, she attributed her talent to God.

Rowe worked with humble materials such as crayon, cardboard, chewing gum, and felt-tip markers, using bright colors and unexpected juxtapositions to create a sense of energy and movement. Her early works involved single figures, but her later drawings combined memories and fantasy into narrative themes of the southern farm—incorporating people, plants, birds, expressive animals and speckled fish. Drawings made during the last years of her life (including *Picking Cotton*) are largely autobiographical. After receiving a diagnosis of multiple myeloma in 1981, Rowe continued to draw until her last months of life. With the assistance of an Atlanta-based art dealer, Rowe’s work gained national recognition. In 1982 (the year of her death), Rowe’s drawings were included in the Corcoran Gallery of Art’s exhibition “Black Folk Art in America, 1930–1980,” which is regarded as the beginning of American recognition of self-taught artists.

According to its owner, William Arnett, *Picking Cotton* (1981) could have been entitled “What I don’t like about my life as I look back on it.” The picking of cotton refers to the most degrading form of manual labor the artist knew. Rowe is seen in a lush field in an orange party dress (a statement that this work was not meant for her), bending over with a tool in her hand and a cotton-sack on her back in the shape of a vulnerable woman. Imposed on the artist, and central to the composition, is a black mule, symbolizing the forced labor of the black woman. The seated white woman scrutinizing Rowe may represent her employers during her years as a domestic servant; the red rodent beneath her chair is Rowe’s symbol for an unpleasant situation. In addition, the blue-green man sitting in the lower left corner, who grins while he watches her work, may represent her former husbands. The picture also includes brightly colored birds with pointed beaks, one pecking from a curved bush whose pointed petals suggest a ravenous set of jaws.

*Picking Cotton*, with its animated and unsettling themes, evokes the complex and ever-changing interactions among people, animals, and the environment that can lead to the emergence or resurgence of human infectious diseases. Many of Rowe’s wild and domestic animals are associated with high-consequence zoonotic pathogens addressed in this issue. For example, her bird pecking at the “jaws of death” could serve as a symbol of the role birds play in spreading West Nile and avian influenza (H7N9) viruses. Her rat brings to mind rodent-borne pathogens, including a novel hantavirus and lymphocytic choriomeningitis virus, and her dogs bring to mind reports of the resurgence of rabies virus.

Rowe’s multifaceted work illustrates her personal mythology, her response to life experiences, and an assimilation of African American spiritual and narrative traditions. The words and drawings of the free-spirited Nellie Mae Rowe—born to a former slave on Independence Day—remind us how much we can learn from people who see things differently, including both artists and scientists.
